# LC-QToF-MS Analysis of Stimulant Drugs and Their Metabolites in Wastewater During Football Games

**DOI:** 10.3390/metabo15020069

**Published:** 2025-01-22

**Authors:** Brandon J. Stamper, Krishna Chaturvedi, Bharathi Avula, Ji-Yeong Bae, Yan-Hong Wang, Kyle S. Bledsoe, Ikhlas A. Khan, Murrell Godfrey

**Affiliations:** 1 Department of Chemistry and Biochemistry, University of Mississippi, University, MS 38677, USA; bjstamper43@gmail.com (B.J.S.); kchaturv@olemiss.edu (K.C.); kbledso1@go.olemiss.edu (K.S.B.); 2 National Center for Natural Products Research, University of Mississippi, University, MS 38677, USA; bavula@olemiss.edu (B.A.); jybae@jejunu.ac.kr (J.-Y.B.); wangyh@olemiss.edu (Y.-H.W.); ikhan@olemiss.edu (I.A.K.); 3 Department of BioMolecular Sciences, Division of Medicinal Chemistry, School of Pharmacy, University of Mississippi, University, MS 38677, USA

**Keywords:** LC-QToF-MS, wastewater, stimulants, drugs of abuse, metabolites, football games

## Abstract

Background: The use of illicit drugs and stimulants is a burgeoning socioeconomic problem, ultimately leading to an increase in street crimes and deteriorating human health. The persistent presence of CNS drugs in wastewater can also lead to downstream adverse effects on aquatic wildlife and humans. Objectives: In the present study, a method was developed for the solid-phase extraction and quantitative liquid chromatography coupled with tandem quadrupole/time-of-flight mass spectrometry (LC-QToF-MS) analysis of CNS stimulant drugs and their metabolites in municipal wastewater. The targeted species included amphetamine, methamphetamine, methylenedioxy-methamphetamine (MDMA), methylenedioxyethylamphetamine (MDEA), cocaine, and benzoylecgonine (BE). Methods: The method was validated and applied to analyzing wastewater samples collected at the University of Mississippi and the City of Oxford wastewater treatment plants (WWTPs) during weekends when the university hosted home college football games. Results: Our results indicate that while amphetamine, methamphetamine, MDMA, cocaine, and BE were all detected at quantifiable levels, amphetamine and BE were present in significantly higher concentrations in wastewater during football games. Conclusion: The insights from this study can be utilized to monitor long-term drug use trends, providing local law enforcement agencies with relevant data on consumption patterns over time.

## 1. Introduction

The use of illicit drugs and stimulants is a growing socioeconomic concern, ultimately resulting in increasing crime rates and deteriorating human health. It is recognized by the European Monitoring Centre for Drugs and Drug Addiction (EMCDDA) that cocaine and marijuana use has increased dramatically since ca. 2003 [1,2,3]. Pal et al. [4] conducted a study on illicit drugs and their environmental impact, concluding that human health and ecosystem functioning can be potentially impacted by drug use, even in low concentrations. The presence of cocaine, morphine, amphetamine, methylenedioxymethamphetamine (MDMA), or a complex mixture thereof, in water may adversely affect aquatic organisms and human health, especially considering the potent pharmacological activities of these drugs [4]. The prospect of using liquid chromatography with tandem mass spectrometry (LC-MS/MS) as a method to determine drug usage range through non-intrusive drug monitoring at sewage treatment facilities was first suggested by Daughton in 2001 [5]. Daughton recommended such a method as the first feasible approach to obtain real-time data that would simultaneously reflect the usage of illicit drugs throughout an entire community and provide environmental data for potential pollutants that were not previously considered [5]. In 2005, Zuccato et al. [6] published research using Daughton’s method in an experiment focusing on the increasing trend of cocaine usage from samples collected at four different sites in Italy and the River Po [6]. Since then, LC-MS, among other techniques, has been used to study sewage-based epidemiology in smaller populations of different countries and has also been used to assess the risks of these drugs and metabolites in environmental water sites after the treatment of wastewater [7].

Weekend increases in stimulant concentrations observed in prior studies often reflect generalized urban trends, which are influenced by diverse populations and activities. For example, Vuori et al. [8] observed statistically significant increases in MDMA and benzoylecgonine concentrations in two out of nine Finnish cities during weekends. However, concentrations of other stimulants, cannabis, and opiates remained relatively constant throughout the week. Similarly, T. Nefau et al. [9] reported regional weekend trends in cocaine and MDMA concentrations in the wastewater of certain French cities, but not for cannabis or opiates. These findings highlight the regional variability and inconsistency of weekend trends across different settings.

**Figure 1:**
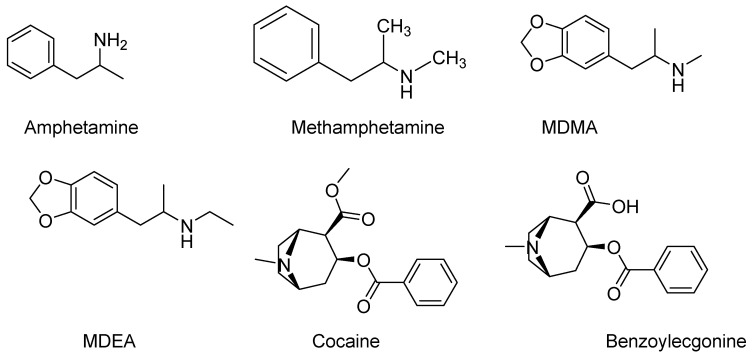
Chemical structures of six drugs and metabolites analyzed in this study.

Recently, the study of sewage-based epidemiology (SBE) and drug utilization trends has concentrated primarily on large social and public events. In 2016, Gul et al. [10,11] conducted a novel analysis of drug trends from wastewater directly obtained from football sporting event venues and the surrounding areas. Since this study analyzed the prevalence of drug abuse at high-population events (i.e., football games) as well as the effects on the surrounding communities and environmental wastewater treatment plants, this study was considered highly relevant to the scientific community. As home collegiate football games at the University of Mississippi (Vaught-Hemingway Stadium, University, MS 38677) averaged an attendance of 59,969 people for 2017, such occurrences could also be considered high-population events with the potential for heightened drug abuse. Therefore, in continuing our studies on wastewater samples collected at the University of Mississippi and in the City of Oxford, this study was developed to analyze the effect of a collegiate football game on the presence of prominent drugs and metabolites of abuse (e.g., cocaine, amphetamine, methamphetamine, methylenedioxy-ethylamphetamine (MDEA), MDMA, and benzoylecgonine (BE)) (Figure 1) in the local population. The above compounds were chosen because of their widespread abuse. Stimulants such as cocaine and amphetamines, including their derivatives and metabolites, are among the most widely used and abused illegal substances in the United States [12]. Amphetamine and its congener, methamphetamine, belong to the phenethylamine class of substances known for their potent central nervous system (CNS)-stimulating activity. It is used for the treatment of attention deficit hyperactivity disorder (ADHD), obesity, and narcolepsy (sleep disorder) [12]. MDEA, a substituted amphetamine, is used recreationally in a similar manner to MDMA (ecstasy) [13]. Because cocaine is a potent and highly addictive CNS stimulant drug, the primary urinary metabolite of cocaine, benzoylecgonine (BE), was logically selected to be monitored [14]. The novelty of our research lies in the application of liquid chromatography quadrupole/time-of-flight mass spectrometry (LC-QToF-MS) for analyzing drugs of abuse in municipal wastewater from a university. Our study uniquely focuses on the prevalence of drug abuse during high-population events, such as collegiate football games, and assesses its impact on the surrounding communities and wastewater treatment plants. This targeted approach provides valuable insight into local drug use patterns during these events. In the present study, we collected wastewater samples from wastewater treatment plants within the City of Oxford, Mississippi, and at the University of Mississippi during home collegiate football games in the autumn of 2017. A liquid chromatography-quadrupole/time-of-flight mass spectrometry (LC-QToF-MS) method was chosen for wastewater analysis due to its dual capability to quantify targeted analytes while simultaneously screen for the accurate mass of untargeted compounds, providing a comprehensive analytical approach.

## 2. Materials and Methods

### 2.1. Sample Collection

The influent wastewater samples were collected from the City of Oxford Wastewater Treatment Plant (WWTP) and the university WWTP (8 and 6 total sample days, respectively) during October 2017. Wastewater samples were collected on Friday before the football game, on Saturday during the game, on Sunday after the game, and on Wednesday for baseline calculations. A total volume of 200 mL for each sample was collected. Game day samples were collected at timepoints two hours before the start of the football game, at the beginning of the game, two hours after the start of the game, and four hours after the start of the game. On days other than game day, the samples were collected at 10:00 A.M., 12:00 P.M., 2:00 P.M., and 4:00 P.M. After collection, the samples were filtered using gravity filtration to remove any solid material and large particles from the samples before extraction and they were subsequently stored at −20 °C before analysis.

### 2.2. Extraction Procedure

Phenomenex Strata Screen-C (55 µm, 70 Å) 150 mg/ 3 mL solid-phase extraction (SPE) cartridges were used for the SPE of wastewater samples. The SPE cartridges were conditioned with 2 mL of methanol followed by 2 mL of deionized water. Drugs and internal standards were placed in glass tubes, and 1 mL of blank wastewater was added into the tubes to create the calibration curves. The calibration curves were prepared for all analytes simultaneously, ranging from 1 ng/mL to 500 ng/mL. The first point of the curve represented the limit of quantitation (LOQ). Each curve was repeated multiple times to ensure reliability, and data below the LOQ and above the upper limit of linearity (ULOL) were excluded. Samples were added by positive pressure on the cartridges. The cartridges were washed with 9 mL of 0.1 N HCl, followed by 6 mL of methanol, and then dried under a vacuum. Analytes were then eluted with 2 mL of 1% ammonia in methanol into glass vials. The vials were then evaporated under nitrogen to a volume of 1 mL, vortexed, and evaporated under nitrogen to dryness. The samples were then rehydrated with 100 micro-liters of 0.5 N HCl in methanol, transferred into inserts, and placed in LC vials for analysis. We used HCl in methanol to dissolve the dry residue because, at this stage, the analytes were in their base forms. Methanol was more effective as a solvent for dissolution, and the addition of HCl facilitated the conversion of the analytes to their hydrochloride forms, enhancing their solubility and stability for subsequent analysis.

### 2.3. Instrumentation and Chromatographic Conditions

#### Liquid Chromatography Quadrupole/Time-of-Flight Mass Spectrometry (LC-QToF-MS)

The liquid chromatographic system was an Agilent Series 1290 (Santa Clara, CA, USA) comprising a binary pump, a vacuum solvent microdegasser, an autosampler with a 100-well tray, and a thermostatically controlled column compartment. Separation was achieved on an Agilent Poroshell 120 EC-8 (2.1 × 100 mm, 2.7 µ) column. The mobile phase consisted of water (A) and acetonitrile (B), both containing 0.1% formic acid at a flow rate of 0.2 mL/min, in the following gradient elution: 0 min, 95% A: 5% B to 77% A: 23% B in next 17 min. Each run was followed by a 3 min wash with 100% acetonitrile and an equilibration period of 3.5 min. Two microliters of sample were injected, and the column temperature was maintained at 37 °C.

The mass spectrometric analysis was performed with a QToF-MS/MS (Model #G6530A, Agilent Technologies, Palo Alto, CA, USA) equipped with an ESI source with Jet Stream technology using the following parameters: drying gas (N_2_) flow rate, 9.0 L/min; drying gas temperature, 250 °C; nebulizer, 30 psig, sheath gas temperature, 325 °C; sheath gas flow, 9 L/min; capillary, 3000 V; skimmer, 65 V; Oct RF V, 750 V; and fragmentor voltage, 125 V. The sample collision energy was set at 35 eV. All of the operations, acquisition, and analysis of data were controlled by Agilent MassHunter Acquisition Software Ver. A.05.00 and processed with MassHunter Qualitative Analysis Software Ver. B.07.00. Each sample was analyzed in positive mode in the range of *m*/*z* = 100–1300. Accurate mass measurements were obtained utilizing reference ion correction using reference masses at *m*/*z* 121.0509 (protonated purine) and 922.0098 [protonated hexakis (1H, 1H, 3H-tetrafluoropropoxy) phosphazine or HP-921] in positive ion mode. The compounds were confirmed in each spectrum. For this purpose, the reference solution was introduced into the ESI source via a T-junction using an Agilent Series 1200 isocratic pump (Agilent Technologies, Santa Clara, CA, USA) using a 100:1 splitter set at a flow rate of 20 µL/min. The selected ions were then submitted to collision with nitrogen in a high-pressure collision cell, and the collision energy (over a gradient from 20 to 80 V) was optimized to afford good product ion signals; subsequently, mass was measured with the ToF analyzer. The instrument was operated in DIA (data-independent acquisition) mode consisting of a full MS survey scan in the 100–1700 Da range (scan time:100 ms) followed by an MS/MS scan for the three most intense ions. The compounds were confirmed in extracted ion chromatogram (EIC) mode. Ions [M+H]^+^ = *m*/*z* 136.1121, 150.1277, 194.1176, 208.1332, 290.1387, and 304.1543 for amphetamine, methamphetamine, MDMA, MDEA, benzoylecgonine, and cocaine, respectively, were selected as ions for detection and were found with less than 5 ppm mass error.

### 2.4. Chemicals

The analytical drug standards and deuterated internal standards for amphetamine, methamphetamine, MDMA, MDEA, cocaine, and BE were purchased from Cerilliant Corporation (Round Rock, TX, USA), from which controls were made. The reference standard (HP-921) was purchased from Lipomed AG (Arlesheim, Switzerland). All solvents (HPLC grade) and reagents were obtained from VWR International (Radnor, PA, USA) and Sigma-Aldrich (St. Louis, MO, USA).

### 2.5. Validation of the Analytical Procedure

The ICH harmonized tripartite guideline was used to validate the analytical procedure [15]. Positive controls were prepared at concentrations of 5 and 10 ng/mL. Analyte concentrations of 5 ng/mL and 10 ng/mL were treated as quality controls (QCs). These concentrations were chosen based on our expectation of the detection levels in wastewater samples, as they are representative of potential environmental concentrations, especially considering that the upper limit of linearity (ULOL) was set at 100 ng/mL or 500 ng/mL. These controls were then extracted and analyzed using the procedure described above. The first point of the curve represented the limit of quantitation (LOQ). Each curve was repeated multiple times to ensure reliability, and data below the LOQ and above the upper limit of linearity (ULOL) were excluded. The limit of detection (LOD), LOQ, and the upper limit of linearity were determined for each drug. Within-batch and between-batch accuracy and precision were determined by analyzing 5 batches, each with 3 replicates at both concentrations.

### 2.6. Sample Analysis

The wastewater samples were extracted and analyzed using the validated procedure. The real wastewater samples were prepared as composite samples containing 2.5 mL of the samples collected at each time for each day. Analyzing single timepoint spot samples can potentially misrepresent the “average” concentration. By creating a composite sample, we aimed to provide a more accurate representation for day-to-day comparisons of the data. A total of 10 mL of sample was extracted from the wastewater samples as a pre-concentration strategy due to the low analyte concentration in the wastewater. Calculations of the final concentration accounted for the pre-concentration of the samples. Statistical analysis was performed by a two-way ANOVA test with a significance level of *p* < 0.05 using GraphPad Prism 9.1 software (GraphPad Software, Inc., San Diego, CA, USA), software.

## 3. Results and Discussion

SPE cartridges were used to extract the samples for method validation. For all of the analytes, their respective deuterated internal standards were used. The deuterated internal standards were used to minimize matrix effects and improve accuracy by co-eluting with the analytes during LC/QToF-MS and correcting for minor ion suppression or enhancement. This method was successfully validated for the analysis of six drugs in the wastewater samples. The concentration–response relationship for all analytes was linear between the concentration and analyte/IS peak area ratio. In addition, all analytes showed r^2^ values greater than 0.999. A spike recovery study showed an accuracy of 95 to 103% with a standard deviation (SD) of 0.18 to 0.32 for 5 ng/mL positive controls and 96 to 102% accuracy with 0.26 to 0.62 SD for 10 ng/mL positive controls. Method LOD, LOQ, and upper limit of linearity (ULOL) varied by analyte and are shown in Table 1. In addition, Table 2 shows the within-batch and between-batch accuracy and precision for each analyte. Finally, this method was validated to show a high degree of accuracy and precision for the analyzed drugs and metabolites.

College football games often draw tens of thousands of attendees per game in the southeastern region of the United States of America. Due to the nature of these events, the attending fans are often inebriated. Consequently, increased drug use has been observed at football games in prior studies [10,11,16,17]. This study aimed to identify the concentrations of drugs and metabolites at typical southeastern collegiate football games using a novel LC-QToF-MS method. The samples collected at the University of Mississippi continued the observed trend of rising levels of amphetamine, cocaine, and benzoylecgonine during home football games. In addition, the samples contained higher concentrations of methamphetamine than previously observed. Furthermore, the methamphetamine concentration was evidenced to significantly increase during the game. While MDMA was identified in two samples, no trends for its presence were observed. The data are summarized and presented in Table 3 and Table 4. Overall, these data indicate that stimulant drug use increases during home collegiate football games at the University of Mississippi.

Based on previous studies, the temporal effects of weekends on drug concentrations in wastewater are inconsistent. Postigo et al. (2011) and Metcalfe et al. (2010) observed only slight increases in cocaine concentrations over weekends, while Huerta-Fontela et al. (2008) found that cocaine levels peaked on Sundays and Mondays but remained relatively constant throughout the week [18,19,20]. Vuori et al. (2014) reported statistically significant weekend increases in MDMA and benzoylecgonine concentrations in two out of nine Finnish cities; however, concentrations of other stimulants, cannabis, and opiates were consistent throughout the week [8].

By contrast, our study demonstrates that stimulant concentrations increased significantly on campus during football game weekends while remaining stable in the surrounding city. This localized pattern likely reflects the sharp influx of visitors onto the campus, with game day attendance averaging ~59,969, compared to a baseline on-campus student population of ~7139 during weekends in 2017. This suggests that event-driven factors, rather than general weekend trends, are responsible for the observed increases.

**Figure 2:**
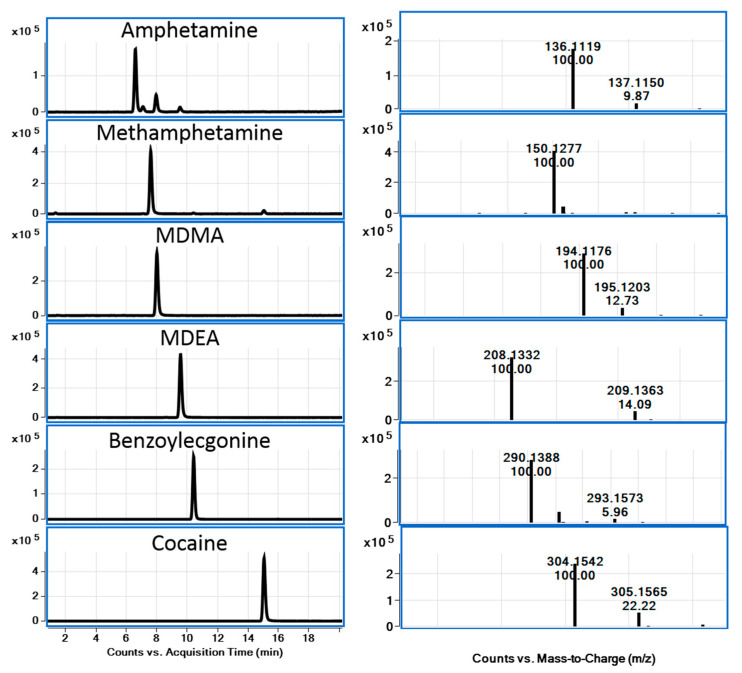
Chromatograms and MS spectra for the six analyzed drugs in spiked wastewater samples.

**Figure 3:**
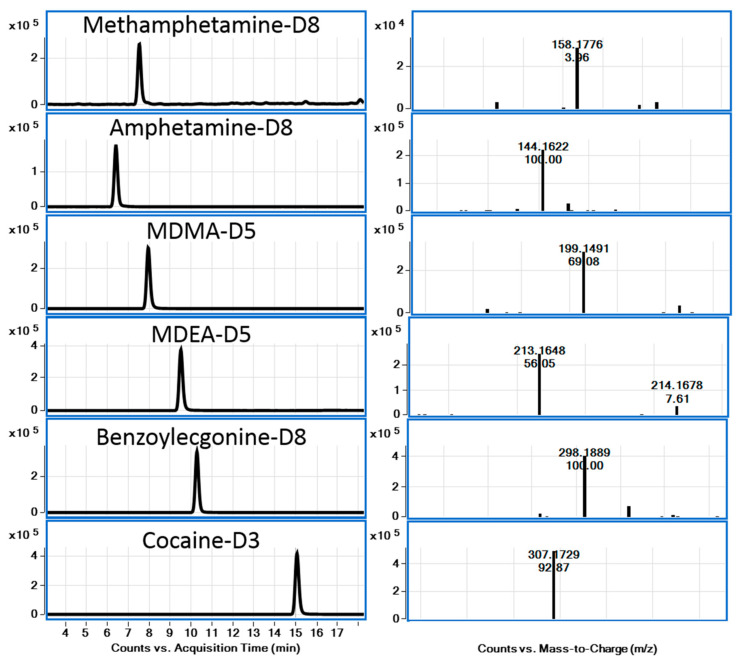
Chromatograms and MS spectra for the six deuterated internal standards in spiked wastewater samples.

**Figure 4:**
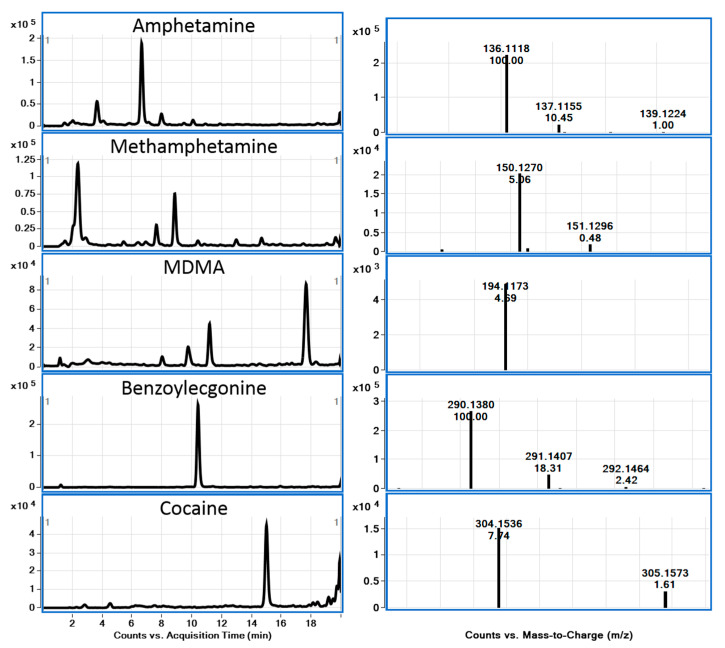
Chromatograms and MS spectra of the five positive drugs in sample C5.

LC-QToF-MS is a powerful technique for identification and confirmation purposes and is also a very attractive tool for the wide-scope screening of drugs of abuse in the environment and in wastewater [21]. Accurate mass spectral data have been obtained using QToF-MS for some classes of illicit drugs, and their metabolites are frequently detected in the environment and in wastewater. Based on these data, fragment ions have been elucidated through accurate mass information, which is imperative for confirming the identity of such fragment ions and obtaining the elucidation of structures of several key ions [22]. All information provided using QToF-MS about fragment ions helps to ensure the identity of precursor ions with high reliability. It cannot be overstated that the knowledge of accurate mass data and fragment ions is highly useful for the recognition of related compounds when developing MS-based analytical methods for trace-level determination in complex matrices. In this work, accurate mass spectra of selected compounds were obtained using LC-QToF-MS. Accurate mass spectra of 6 drugs of abuse and related compounds, including two metabolites and deuterated analogs, were studied in this work (Figure 2, Figure 3 and Figure 4).

From the spectra, the MS/MS fragment ion of the [M+H]^+^ ion of amphetamine and methamphetamine was found to be similar, with *m*/*z* 119.0847, resulting from the loss of ammonia or methylamine. MDMA and MDEA, containing a dioxole ring, gave similar fragment ions. As an example, MDMA ([M+H]^+^ with *m*/*z* 194.1176) showed a loss of CH_3_NH_2_ to a fragment ion with *m*/*z* 163.0753 and β-C-C cleavage, leading to a 1,3-benzodioxol-5-yl methylium ion with *m*/*z* 135.0451 due to the loss of ethylamine. This pattern was similar to amphetamine and related compounds. A major fragment ion with *m*/*z* 105.0704 [C_8_H_9_]^+^ was interpreted due to the loss of H_2_C=O from the ion with *m*/*z* 135.0451. Cocaine and its major metabolite, benzoylecgonine, are frequently reported to be found in the aquatic environment. The most abundant fragment ion of cocaine and its metabolite can be assigned to the neutral loss of benzoic acid (122 Da), resulting in a fragment ion with *m*/*z* 182.1171 for cocaine. The fragment ion with *m*/*z* 168.1010 for benzoylecgonine involved a further loss of methanol or water (*m*/*z* 164.1080 and 150.0910 for cocaine and benzoylecgonine, respectively).

As home football games attract large numbers of fans, these games have significant impacts on the surrounding community. Visiting game attendees are a boon for local businesses, such as hotels, bars, and restaurants. Students and local residents are also encouraged to attend restaurants, bars, and parties more frequently. Additionally, many people watch the game from their homes or at sports bars. Our analyses show that drug concentrations in the City of Oxford’s wastewater are unaffected during football games. Interestingly, benzoylecgonine concentrations show an increase on the Sundays following home football games, continuing the trend previously reported by Gul et al., 2016 [11]. Moreover, amphetamine, methamphetamine, MDMA, cocaine, and benzoylecgonine were all observed. Our prior studies did not indicate an increase in stimulant drug presence in wastewater on weekends during which a home football game did not occur [17]. This supports a correlation between the increase in drug concentration and game day [23]. It is conceivably possible that the first void on Sunday morning impacts Sunday samples in the City of Oxford and that cocaine consumption is highest at night following home football games. A short-term overnight time-dependent study would provide more insight into this phenomenon.

Our study provides novel insights into the effects of mass gathering events on localized drug use patterns in a collegiate setting. Unlike prior studies that report consistent weekend increases across urban regions [18,19,24], our findings show that stimulant concentrations increased significantly on campus during football game weekends while remaining relatively stable in the surrounding city. This suggests that the observed patterns are driven by the unique event-driven influx of individuals onto the campus rather than general weekend behavior. The differences in scale, population density, and setting further distinguish our findings from those reported in broader urban studies.

## 4. Conclusions

An LC-QToF-MS method was developed and successfully validated for the analysis of stimulant drugs in municipal wastewater. The method was accurate and reproducible for all six analytes. The method was then applied for the analysis of amphetamine, methamphetamine, MDMA, MDEA, cocaine, and benzoylecgonine in samples collected at the University of Mississippi and in the City of Oxford during home football games at the university. The results revealed increasing concentrations of four stimulant drugs in the university’s wastewater during the games. These results also verified previously published LC-MS/MS data [10]. This trend was prevalent for amphetamine, methamphetamine, and cocaine and its metabolite, benzoylecgonine, suggesting that amphetamine, methamphetamine, and cocaine are being self-administrated by football game attendees. This trend is likely consistent with other large universities, but further analyses are needed to quantify these drugs at different locations.

Such stimulant drugs are more prominent during football games because they increase the heart rate and boost short-term energy. For fans administering these drugs, the result is increased excitement during the game, at the cost of potential long-term cardiovascular damage [25,26]. Other adverse effects of stimulant drugs are agitation/belligerency or other more severe short-term effects, especially when used in conjunction with alcohol. Notably, the City of Oxford’s wastewater exhibited increases in benzoylecgonine on days following home football games. This may be the result of consumption of cocaine on the prior day (game day). Nevertheless, previous studies of cocaine metabolism in urine showed that the excretion of cocaine metabolites is typically highest at the first collection time and drastically drops during the first 24 h after consumption [27]. Since game day cocaine consumption should yield higher concentrations on the game day and not the following day, the results suggest that the consumption observed by this study occurs either in the nighttime after the game or on the following day. We also hypothesize that the high signal of native cocaine could be attributed to discarded cocaine or the washing of contaminated materials, rather than solely from metabolic elimination. This possibility highlights the complexity of wastewater systems, where non-consumptive sources may contribute to the presence of drugs [28]. Further investigation is required to determine the actual cause of this increase since our study focuses on a specific collegiate campus environment during football game weekends, where the observed patterns are driven by a sharp influx of visitors rather than routine weekend behavior. This distinction highlights the unique localized nature of our findings, which differ significantly from the broader trends reported in urban wastewater studies. Additionally, the observed stability of stimulant concentrations in the surrounding city underscores the localized impact of these mass gathering events on campus drug use patterns. The insight gained from this study can be used to monitor long-term drug use trends to give local law enforcement agencies relevant data about changes in consumption. A sudden permanent increase may mean a significant change in the local drug trade.

## Figures and Tables

**Table 1:** Method LOD, LOQ, ULOL, and r^2^ for stimulants detected by LC-QToF-MS.

Analyte	LOD (ng/mL)	LOQ (ng/mL)	ULOL (ng/mL)	Correlation Coefficient (r^2^)
Amphetamine	1	2.5	500	0.9996
Methamphetamine	1	2.5	100	0.9996
MDMA	1	5	500	0.9999
MDEA	1	2.5	500	1.0000
Cocaine	1	2.5	100	0.9999
Benzoylecgonine	1	5	100	0.9996

**Table 2:** Within-batch and batch-to-batch accuracy and precision.

Analyte (10 ng/mL)	Within-Batch Accuracy	Within-Batch %CV	Batch-to-Batch Accuracy	Batch-to-Batch %CV
Amphetamine	95–104%	2.7–9.0%	99%	6.2%
Methamphetamine	98–101%	1.1–4.4%	99%	2.7%
MDMA	95–104%	1.9–6.6%	99%	4.5%
MDEA	91–100%	1.6–5.5%	96%	3.7%
Cocaine	95–100%	2.2–8.4%	98%	4.4%
Benzoylecgonine	95–109%	0.9–6.5%	102%	4.1%

*n* = 3 independent experiments.

**Table 3:** Concentrations of drugs and metabolites (ng/mL) in the City of Oxford samples.

Date/Day of Week (Year 2017)		Amphetamine	Meth	MDMA	MDEA	Cocaine	BE
20-October	Fri	1.0 *	0.1	-	-	0.2	1.8 *
21-October	Sat	0.9	0.3	<LOQ	-	0.3	3.7 *
22-October	Sun	0.8	0.1	-	-	0.2	3.8 *
25-October	Wed	0.7	0.2	-	-	0.1	1.2
28-October	Sat	0.6	0.2	0.1	-	0.3	1.4
29-October	Sun	0.6	0.3	-	-	0.2	3.3 *

* *p*-value < 0.05 considered statistically significant; *n* = 3 independent experiments.

**Table 4:** Concentrations of drugs and metabolites (ng/mL) in the University of Mississippi samples.

Date/Day of Week (Year 2017)		Amphetamine	Meth	MDMA	MDEA	Cocaine	BE
13-October	Fri	1.2	0.1	-	-	0.1	0.6
14-October	Sat	2.1 *	0.2	<LOQ	-	0.2	1.8 *
20-October	Fri	1.1	0.1	0.2	-	0.1	0.6
21-October	Sat	2.9 *	0.2	0.1	-	0.2	3.2 *
22-October	Sun	0.1	-	<LOQ	-	-	0.8
25-October	Wed	2.1 *	-	-	-	-	0.4
28-October	Sat	2.1 *	0.3	<LOQ	-	0.1	1.4 *
29-October	Sun	0.4	0.4	-	-	-	0.6

* *p*-value < 0.05 considered statistically significant; *n* = 3 independent experiments.

## Data Availability

Data are contained within the article.
